# Guidelines for Comparing Circular Dichroism Spectroscopy and Molecular Dynamics Simulations for Biomolecules

**DOI:** 10.1002/cphc.202600010

**Published:** 2026-06-23

**Authors:** Cinzia Campus, Marta Roldo, Charles H. Chen, Roja Hadianamrei, Elisabetta Gavini, Christian Jorgensen

**Affiliations:** ^1^ Department of Chemical, Physical, Mathematical and Natural Sciences Sassari Italy; ^2^ School of Medicine Pharmacy and Biomedical Sciences University of Portsmouth Portsmouth UK; ^3^ Synthetic Biology Group Research Laboratory of Electronics Massachusetts Institute of Technology Cambridge Massachusetts USA; ^4^ Department of Medicine, Surgery and Pharmacy Sassari Italy

**Keywords:** blood–brain barrier, circular dichroism, drug delivery, endothelial cell membrane, molecular dynamics, peptide drug delivery

## Abstract

We consider a combination of circular dichroism (CD) spectroscopy and molecular dynamics (MD) simulations to study peptide partitioning through biological membranes. We discuss the main equations involved in both methods, including their caveats and practical limitations. We then proceed to outline a set of recommendations for how to best compare CD to MD data sources, including methodological limitations. We envisage that this work can serve as a reference source for community members seeking to glean insights via a combination of CD and MD methods.

## Graphical abstract

A direct comparison is made between circular dichroism (CD) spectroscopy and molecular dynamics (MD) simulations for studying peptide partitioning through biological membranes. The assumptions and limitations of each method is discussed, and case studies are provided for their use.

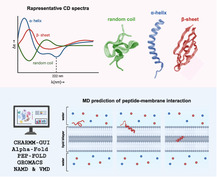

## 1 Introduction

Peptides capable of crossing complex biological barriers, such as the blood–brain barrier (BBB) or bacterial membranes, are becoming increasingly important due to their therapeutic potential in precision medicine applications. For example, antimicrobial peptides are promising candidates in the fight against drug‐resistant bacterial infections [1]. Molecular dynamics (MD) simulations offer detailed mechanistic insights into peptide–membrane interactions, but they require experimental validation to ensure predictive reliability. To improve the accuracy of MD predictions and deepen our understanding of peptide insertion, conformational dynamics, and lipid perturbation, we propose circular dichroism (CD) spectroscopy as a complementary experimental probe. This combined approach provides a simple but powerful experimental toolkit for investigating peptide interactions in complex, barrier‐relevant systems. We outline a roadmap highlighting the strengths, limitations, and synergies of MD and CD spectroscopy, with the goal of advancing both mechanistic understanding and the rational design of peptides optimized for barrier penetration.

## 2 Therapeutic Delivery of Peptides

The delivery of therapeutic peptides requires understanding the underlying intrinsic biophysical and biochemical characteristics of the therapeutic, in particular the interactions of the therapeutic with the biological barrier to be crossed [2], as well as absorption, distribution, metabolism, excretion challenges and intrinsic permeability [3]. Although peptides offer high potency and selectivity, their clinical translation is often limited by poor bioavailability, low absorption, and rapid degradation in vivo [2, 4, 5]. Due to their conformations and physicochemical properties, peptides are highly susceptible to enzymatic breakdown by proteases, peptidases, and even enzymes such as cytochrome P450 in mucosal tissues [4, 5, 6]. When administered orally, they face additional challenges, including degradation in the acidic environment of the stomach and by gastrointestinal enzymes, resulting in minimal systemic absorption [2, 4, 6]. Their generally hydrophilic nature and relatively high molecular weight (>500 Dalton) also limit their ability to permeate cellular membranes and other biological barriers [2]. These factors contribute to poor metabolic stability, a short duration of action, and rapid clearance from circulation [4, 7]. The challenge is even more pronounced in targeting the central nervous system, where the BBB, reinforced by efflux mechanisms such as P‐glycoprotein (P‐gp), severely limits the peptide entry into the brain. Together, these obstacles limit the therapeutic potential of peptides, often necessitating parenteral routes of administration, which in turn reduce patient compliance and clinical utility [2, 4].

**FIGURE 1:**
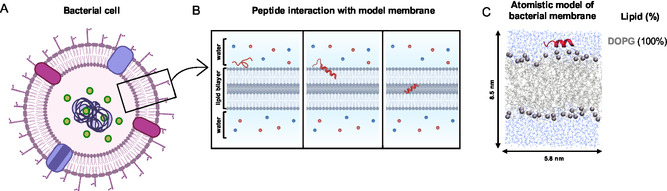
System example: peptide interacting with a membrane. (A) Schematic of a bacterial cell highlighting the lipid bilayer and internal components, with a region of interest indicated for peptide interaction studies. (B) Cross‐sectional illustrations of a model membrane showing red helical peptide at different stages of interaction: approaching, partially inserted, and fully associated with the lipid bilayer. (C) Atomistic model of a bacterial membrane composed of 100% 1,2‐dioleoyl‐sn‐glycero‐3‐phosphoglycerol (DOPG) lipids, with an embedded peptide, illustrating the molecular‐level detail used by MD simulations to study molecule insertion and interactions. Created in https://BioRender.com.

In Figure 1, we present a schematic understanding of therapeutic delivery to the bacterial cell (Figure 1A). The peptide needs to partition through the membrane wall, which requires adequate sampling to observe in a simulation and the likelihood of it happening can be increased by tuning the peptide sequence and structure (Figure 1B). To model such a process, we need to better understand the membrane models at large. For example, we consider an atomistic model of a bacterial membrane composed of 100% 1,2‐dioleoyl‐sn‐glycero‐3‐phosphoglycerol (DOPG) lipids (Figure 1C), illustrating the molecular‐level detail used by MD simulations to study molecule insertion and interactions.

It should be noted that peptides penetrate a number of important complex biological barriers besides the bacterial cell membrane, including mitochondrial membrane [8, 9], the endothelial cell membrane of the BBB [10, 11, 12], the nose‐to‐brain barrier [4], as well as the *Stratum Corneum* [13, 14], although the latter is generally regarded as a highly challenging pathway for delivery [15].

## 3 Circular Dichroism

**FIGURE 2:**
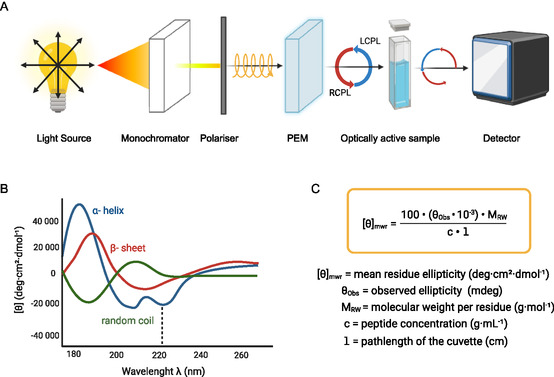
Overview of CD spectroscopy. (A) Components in the CD spectroscopy process; a nonpolarized light source is put through a monochromator, like a grating or prism setup, which polarizes the light. Then, it is passed through a PEM to precisely modulate light polarization into rapidly alternating left and right CPL. The light then passes through the quartz cuvette containing the lipid membrane and the peptide in solution. The diffracted light source is then detected as a signal [17]. Created in https://BioRender.com (B) Representative CD spectra of different protein secondary structures [18, 19]. The figure illustrates the typical signatures of α‐helical (blue), β‐sheet (red), and random coil (green) conformations. The α‐helix profile displays a positive band near 190 nm and two negative minima around 208 and 222 nm, while the β‐sheet spectrum shows a positive band near 195 nm and a negative band around 215–218 nm. The random coil profile is characterized by a strong negative band near 195–200 nm. The dashed line at 222 nm indicates a commonly used reference wavelength for estimating α‐helical content. (C) Equation used for the calculation of mean residue ellipticity ([*θ*]_mwr)_ from the experimentally observed CD signal. The observed ellipticity ([*θ*]_Obs)_ is converted to degrees and normalized by the peptide concentration (*c*), optical path length of the cuvette (*l*), and residue molecular weight (*M*_RW_), yielding the mean residue ellipticity [*θ*]_mwr_ expressed in deg·cm^2^·dmol^−1^.

CD spectroscopy is recognized as the primary experimental technique for characterizing the secondary structure and conformational dynamics of peptides, a process fundamentally critical to membrane interaction studies [16]. The technique is based on the differential absorption of left‐ and right‐circularly polarized light (CPL) by chiral molecules. When CPL passes through an optically active sample such as a peptide or protein, the difference in absorption between the two polarizations, expressed as ellipticity, produces a characteristic CD spectrum. This spectrum provides information on the overall folding and secondary structural content (α‐helix, β‐sheet, random coil) of the molecule. In Figure 2A, we depict the workings of CD spectroscopy. Light from a broadband source is first passed through a monochromator to select a specific wavelength and then through a photoelastic modulator (PEM), which converts linearly polarized light into CPL [20, 21]. When this polarized light passes through a chiral sample such as a protein solution, left‐ and right‐handed CPL are absorbed to different extents, producing a measurable CD signal that is detected as ellipticity. In the far‐UV region, CD spectra are particularly sensitive to protein secondary structure, displaying characteristic patterns that distinguish α‐helices, β‐sheets, and random coils (Figure 2B) [22]. To enable comparison between samples (Figure 2C), the main working parameter in CD spectroscopy is the mean residue ellipticity, [*θ*] or [*θ*]_mwr_. This is derived from the observed ellipticity *θ*_Obs_, normalized by sample concentration (*c*), optical path length (*l*), and residue molecular weight (*M*_RW_) which is calculated by dividing the peptide's molecular weight by the number of amino acid residues. This normalization allows direct comparison of CD spectra obtained from samples with different parameters.

Mean residue ellipticity is calculated according to Equation (1)

(1) $${\left[\theta \right]}_{\mathrm{mwr}}=\frac{100\cdot \left(\theta_{\mathrm{Obs}}\cdot 10^{-3}\right)\cdot M_{\mathrm{RW}}}{c\cdot l}$$

where *θ*_Obs_ is the observed ellipticity in millidegrees (mdeg), with the factor 10^−3^ converting millidegrees to degrees; *M*_RW_ is the residue molecular weight (g·mol^−1^); *c* is the protein concentration (g·mL^−1^); and *l* is the optical path length (cm). The factor 100 is the unit‐conversion constant used to ensure that the resulting values of mean residue ellipticity are expressed in deg·cm^2^·dmol^−1^. Note, in the literature, [*θ*] or [*θ*]_mwr_ is used interchangeably and not always in a consistent manner.

CD is particularly valuable because many therapeutic peptides, such as antimicrobial peptides (AMPs), typically exist in an unfolded random coil structure in aqueous solution, a conformation characterized in the CD spectra by a negative peak near 198–200 nm [16, 23, 24]. Upon interaction with the hydrophobic environment of biological membranes or membrane mimetic systems, these peptides frequently undergo a critical structural rearrangement, folding into ordered, bioactive structures most commonly into the α‐helix through hydrogen bond formation between the amide group and the carbonyl group of the peptide backbone [16, 23, 24]. This conformational transition is clearly captured in CD spectra by the emergence of two characteristic negative bands at 208 nm (*π*→π*) and 222 nm (*n*→π*) and one characteristic positive band at 191–193 nm (*π*→π*), and can be sampled by the ellipticity parameter [*θ*] [18, 19, 23].

To assess the cell selectivity and membrane affinity of peptides, CD measurements are commonly performed using different lipid model systems: neutral small unilamellar vesicles (SUVs), simulating normal mammalian cell membranes where peptides generally remain unstructured, versus negatively charged SUVs or sodium dodecyl sulfate (SDS) micelles, which mimic microbial or cancer cell membranes [23, 24]. However, membrane curvature and surface tension can be the concerns of SUVs and SDS micelles; therefore, more realistic models have been developed such as large unilamellar vesicles, giant unilamellar vesicles (GUVs), and nanodisks [25, 26]. The extent of this folding can be quantified by measuring the mean residue molar ellipticity at 222 nm, which correlates directly with the peptide's α‐helical content. Such analyses are crucial for establishing structure–activity relationships. Usually, the structural changes are often promoted by electrostatic interactions between the cationic molecules and the anionic lipid headgroups, and the quantified ellipticity in the anionic environment shows a direct correlation with the peptides resulting bioactivity and selectivity against target cells [23, 27].

## 4 Molecular Dynamics

### 4.1 Molecular Dynamics Simulations of Peptides

MD simulations represent a powerful computational complement to experimental methods such as CD, functioning as a “computational microscope” that offers atomic‐level insights into the dynamic and transient processes involved in peptide–membrane interactions [28, 29, 30, 31]. These processes, such as adsorption, insertion, translocation, lysis, and self‐assembly, are often difficult to characterize completely using experimental methods, due to the fluidic nature of the lipid bilayer [28]. Furthermore, computational workflows now allow for the accurate integration of posttranslational modifications like glycosylation, which are critical for realistic membrane modeling [32]. For this reason, MD is widely used to describe the mechanism of action of membrane‐active peptides (MAPs) [33, 34].

The MD system components include a water box, a biological membrane bilayer, background ions, and the peptide. To build such an assembly, there are various options to use, with CHARMM‐GUI [35] and MemGen [36] among the more popular choices. The initial peptide coordinates can be built from a variety of resources, including AlphaFold [37] and PEP‐FOLD [38]. The assembly of the peptide can be done either manually, with Packmol [39], or through one of the GMX tools in GROMACS [40]. Once simulations are complete, the analysis of trajectories is simplified by specialized software such as the Python toolkit LiPyphilic, which automates the characterization of complex membrane dynamics [41].

**FIGURE 3:**
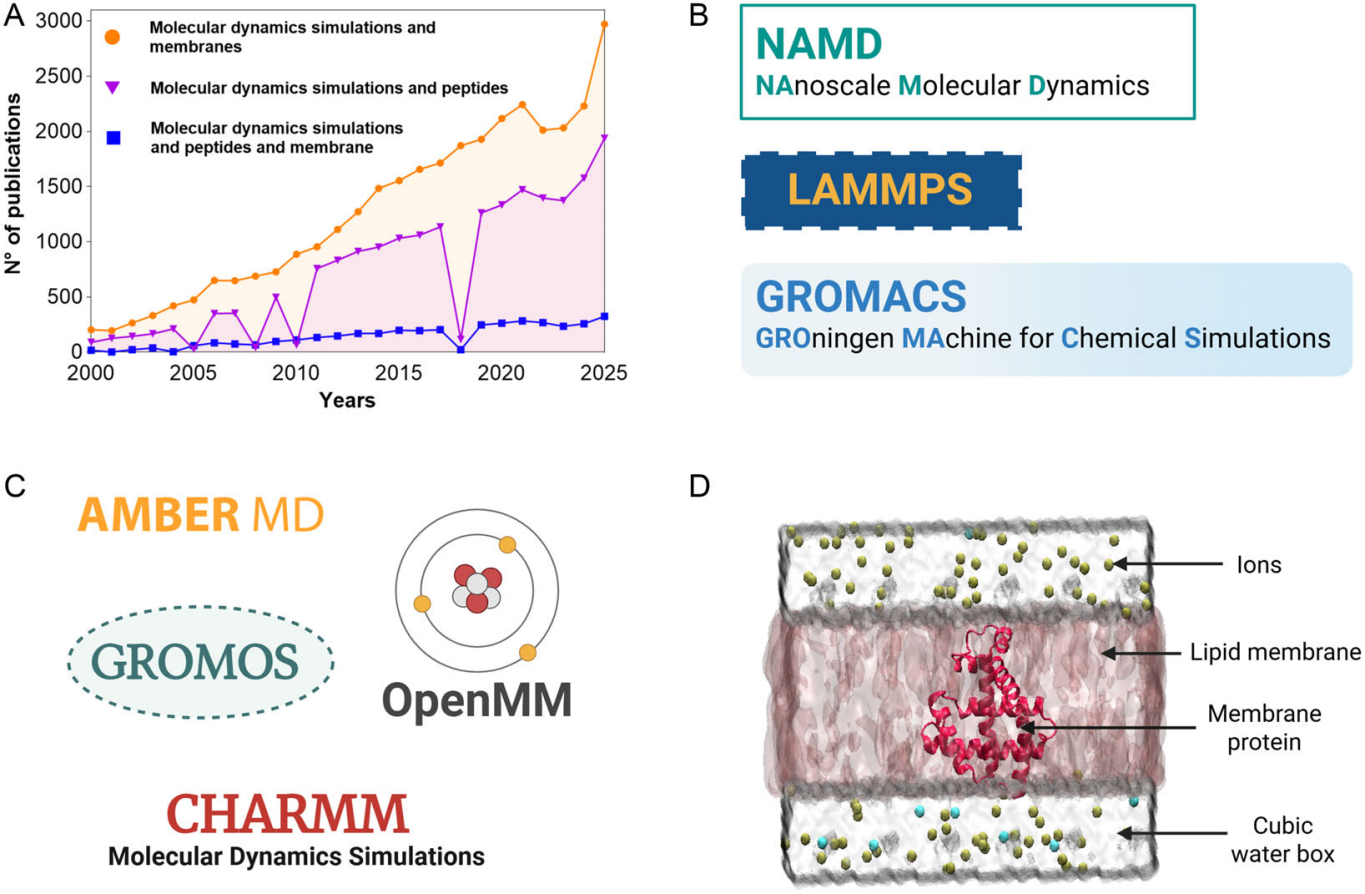
Overview of MD in the study of peptide–membrane systems. (A) Annual number of PubMed‐indexed publications (2000–2025) related to peptide–membrane simulations. This figure shows the yearly number of publications indexed in PubMed from 2000 to 2025 for selected search terms relevant to peptide–membrane simulation studies. The literature search was performed using the following keyword combinations: “*molecular dynamics simulations and membrane,*” “*molecular dynamics and peptides,*” and “*molecular dynamics and peptides and membrane.*” Overall, the data provide an approximate measure of research activity and trends in computational and biophysical investigations of peptide–membrane systems over time. (Source: https://pubmed.ncbi.nlm.nih.gov/) (B) Examples of widely used MD software. (C) Common FFs and software utilized for modeling atomic interactions. (D) Typical MD simulation box setup. The visual highlights the structural hierarchy of the system: a central protein–lipid complex immersed in an explicit water box, with ions included to establish a realistic biophysical environment. Created in https://BioRender.com.

To better understand the utility of MD simulations in peptide partitioning, we proceed to discuss the key equations involved in this MD algorithm, but this has also been discussed in more detail elsewhere [42, 43]. MD simulations generate trajectories of the biological system in time by integrating Newton's second equation of motion (Equation (2)) in time. In practice, this process can only be done according to the slowest degree of freedom (C–H vibrations), so the timestep of integration, *τ*, is typically 2 fs

(2) $$F=\nabla U=m\frac{dx}{dt}$$

where *F* is the force on the atom, ∇U is the derivative of the potential energy, *m* is the mass of the atom, *x* is the position of the atom, and d*x*/d*t* is the time derivative of the position, which is equal to the velocity of the atom. Initially, at *t* = 0, the system is randomly thermalized according to a Maxwell–Boltzmann distribution. Every atom is assigned a random velocity vector, and the atoms are then moved one timestep further, according to Equation (2). To do this, the potential energy *U* of the entire simulation system is calculated through a force field (FF) model, and the derivative of this energy is directly linked to the new positions of the atoms. The most common MD simulation engines include GROMACS [40], NAMD [44], LAMMPS [45], and OpenMM [46], among others. (Figure 3B).

### 4.2 Molecular Dynamics Simulations of Peptide Translocation Across Membranes

MD simulations can directly capture the spontaneous process of a peptide interaction with the membrane [30, 47], including its folding, binding, insertion, and oligomerization into structural assemblies with atomic detail. (Figure 3D) This computational approach builds upon foundational experimental insights such as early studies determining the structure and orientation of mammalian antibacterial peptide, which provided the biophysical basis for understanding the subsequent MD‐derived mechanisms of membrane interaction [48]. This capability allows researchers to precisely quantify folding–partitioning kinetics and determine membrane transfer free energies [49]. In particular, with MD studies, pore and channel formation mechanisms can be elucidated. MD is essential for predicting and validating the 3D structures and dynamic behavior of transmembrane channels and pores. Simulations modeled the Protegrin‐1 (PG‐1) channels, predicting them to be composed of 10 β‐hairpins assembled into an annular shape in an anionic bilayer made up of 1,2‐dioctadecenoyl‐sn‐glycero‐3‐phosphoserine (DOPS) and 1‐palmitoyl‐2‐oleoyl‐sn‐glycero‐3‐phosphoethanolamine (POPE) [50]. Furthermore, MD revealed that some AMPs, such as maculatin, do not form a single stable channel upon partitioning into the membrane, but rather a dynamic ensemble of structurally diverse peptide pores that continually assemble and disassemble [28]. This approach can also guide the rational peptide design. MD simulations provide the instantaneous structural and dynamic information needed for simulation‐guided rational de novo peptide design. By analyzing the ensemble of peptide conformations formed in the membrane, researchers can guide subsequent mutations to engineer properties like oligomerization and potent pore formation, as demonstrated in the stepwise design of the LDKA peptide [33, 51]. MD simulations have also been used to study the mechanism of active transport across membranes. Small peptides are known to use mammalian proton‐coupled peptide transporters, whose substrates are small peptides that bind and are subsequently translocated across the cell membrane [52].

As shown in Figure 3A, publications involving MD simulations of membranes, peptides, and combined peptide–membrane systems have increased over the last two decades, reflecting the growing role of computational methods in understanding biomolecular interactions at membrane interfaces. MD simulations have been used in studying the atomic mechanism of the translocation of highly charged hydrophilic peptides across the hydrophobic core of the cell membranes. Simulations of the HIV‐1 TAT peptide proposed that the strong attraction of the peptides to lipid phosphate groups initiates the insertion of charged arginine side chains, which then nucleates the formation of a transient pore, allowing the peptide to translocate by diffusing along the pore surface. Second, MD simulations have looked at the role of the cell membrane composition to peptide permeation and its relationship to the peptide sequence [31]. For the peptide CM15, simulations confirmed that binding and insertion are significantly stronger in the anionic 1‐palmitoyl‐2‐oleoyl‐sn‐glycero‐3‐phosphoglycerol (POPG) to 1‐palmitoyl‐2‐oleoyl‐sn‐glycero‐3‐phosphocholine (POPC) mixed bilayer (POPG/POPC) model, which mimics the bacterial membrane, compared to the zwitterionic mammalian‐mimic POPC membrane, which the authors attributed to favorable electrostatic interactions [49]. Similarly, simulations of Polymyxin B1 provided insights into its interaction with complex *E. coli* outer and inner membrane models, predicting that while it aggregates in the outer membrane's region, it readily inserts into the inner membrane core, leading to destabilization [53].

### 4.3 Measuring Ellipticity with Molecular Dynamics Simulations

Once the MD simulation of the peptide interacting with the membrane has been completed, the resulting binary trajectory can be visualized with codes such as VMD [54] or PyMOL [55]. As we explained in the CD section, [*θ*] is the mean ellipticity (deg·cm^2^·dmol^−1^), and [*θ*]_222_ is the mean ellipticity at 222 nm (deg·cm^2^·dmol^−1^). From Figure 2B, we see that [*θ*]_222_ is the mean ellipticity for the α‐helical component of the system. According to Hirst (1994), the mean ellipticity at 222 nm, ${\left[\theta \right]}_{222}$, can be calculated using *gmx helix*, as shown in Equation (3) [56]

(3) $${\left[\theta \right]}_{222}=\frac{V_{\infty }}{N_{r}}\sum_{i=1}^{N_{h}}w_{i}\left(r_{i}-k\right)$$

Here, $V_{\infty }$ is the mean ellipticity per residue of an infinite helix; $N_{r}$ is the number of residues of the peptides; $w_{i}$ is the binary ellipticity parameter, which is equal to 1 if analyzing an α‐helix, and 0 if not α‐helix; $r_{i}$ is the length of the helix; k is an experimentally set constant that accounts for terminal effects in α‐helices. It is typically set to k=0 when every helical residue contributes fully to the ellipticity, including residues at the helix termini, while it is set to k≈2.5–3 when the user wants to account for the effective loss of a few residues at the ends of each helix [56].

The origin of Equation (3) can be found in early empirical and theoretical studies that established a quantitative relationship between the CD signal at 222 nm and the α‐helical content of polypeptides. Pioneering work by Hirst and colleagues (1994) employed semiempirical treatments of the peptide amide chromophore to demonstrate that the mean residue ellipticity in the 220–222 nm region is dominated by the *n*→π* transition characteristic of α‐helical structures, and that this ellipticity scales approximately with the number of residues adopting an α‐helical conformation within a molecular ensemble [56, 57]. These analyses showed that ellipticities computed from explicit chromophore models correlate with experimentally determined ellipticity, thereby providing a physical basis for interpreting far‐UV CD minima in terms of helical content. Building on this spectroscopic framework, the Hirst and Brooks model (1994) combined MD simulations with structural analysis to demonstrate that reductions in [*θ*]_222_ observed during protein unfolding correlate with loss of α‐helical structure, and that dynamic effects, such as helix fragmentation and conformational heterogeneity, must be considered when relating CD intensity to helical fraction [56]. Together, these studies support the hypothesis that [*θ*]_222_ is approximately proportional to the number of helical residues, an assumption commonly used in both experimental CD analysis and MD postprocessing [58].

Despite its extensive use, Equation (3) has several limitations that should be considered when interpreting ellipticity estimates derived from MD simulations. The formulation relies on a binary classification of secondary structure, in which residues are designated as either helical or nonhelical, thereby ignoring partially formed, distorted, or transient α‐helical conformations that frequently occur in dynamic peptide–membrane systems [59]. In addition, the use of a constant mean residue ellipticity for an infinite helix (*V*∞) assumes idealized α‐helical behavior and does not account for sequence‐dependent effects or environmental influences such as membrane proximity and heterogeneous solvation, both of which are known to modulate experimental CD signals. The assumption of *k* = 0 further neglects length corrections, potentially leading to an overestimation of ellipticity in short peptides.

The ellipticity of the peptide is influenced by the choice of FF model (Figure 3C). Common successful FFs for peptide include CHARMM [60] and AMBER [61, 62], and we direct the user to comparison studies on the advantages of the respective FFs [63, 64, 65].

The estimation of ellipticity from MD‐derived secondary structure content is semiempirical. Although such approaches are widely used to relate simulated α‐helical content to experimental CD signals, they rely on simplified assumptions regarding the relationship between structural descriptors and spectroscopic observables, and therefore provide only an approximate framework for qualitative comparison with experiment. Accordingly, MD‐derived estimates of [*θ*]_222_ should be interpreted as qualitative indicators of helical propensity rather than quantitatively predictive CD spectra.

### 4.4 Helicity‐Based Secondary Structure Analysis

While ellipticity is an optical signal, helicity is the underlying geometric cause. Helicity is defined as the structural fraction of the peptide that adopts α‐helical conformation, determined by specific backbone dihedral angles, *φ* and *ψ*, and the formation of hydrogen bonds.

Helicity is commonly calculated using the define secondary structure of proteins (DSSP) algorithm in GROMACS [40]. This method assigns a secondary structure classification to each residue on a frame‐by‐frame basis [66, 67]:

gmx do_dssp ‐f trajectory.xtc ‐s topol.tpr ‐o ss.xpm (Command 1)

Here, ‐f trajectory.xtc specifies the trajectory; ‐s topol.tpr specifies the simulation input structure; and ‐o ss.xpm produces a matrix file representing the secondary structure assignments for every residue across the entire simulation time.

By calculating the percentage of time a residue spends in a helical state, empirical relations can then be applied to estimate the expected experimental signal. This distinction ensures that the structural stability observed in the MD trajectory is correctly represented by the macroscopic observations in CD spectroscopy.

### 4.5 Estimation of Mean Residue Ellipticity from Molecular Dynamics Trajectories

To quantify the structural properties of the peptide, standard command‐line procedures within the GROMACS environment are usually employed. A primary objective is to bridge the gap between the simulated ensemble and experimental results by estimating ellipticity. Ellipticity is the optical phenomenon measured via CD spectroscopy. It describes the differential absorption of left and right CPL by chiral molecules. In protein studies, the mean residue ellipticity at 222 nm [*θ*]_222_ serves as a spectroscopic fingerprint for the presence of α‐helical structures.

To analyze the estimated ellipticity, the following command is used:

gmx helix ‐f trajectory.xtc ‐s topol.tpr ‐n index.ndx ‐o cd222.xvg (Command 2)

Here, ‐f trajectory.xtc specifies the simulation trajectory file; ‐s topol.tpr specifies the topology input file; ‐n index.ndx allows selection of the peptide residues if needed; and ‐o cd222.xvg is the output file containing estimated ellipticity values.

It is important to note that these values are derived via an empirical linear transformation of the geometric helicity detected in the MD snapshots, rather than a first‐principles spectral calculation. These analyses provide a robust quantification of the α‐helical fraction, which serves as the structural basis to estimate the mean residue ellipticity at 222 nm. By distinguishing between these geometric and spectroscopic parameters, we can compare MD structural ensembles with experimental CD data [40].

### 4.6 Ab Initio Methods for Circular Dichroism Spectrum Prediction

For high‐fidelity validation, full quantum mechanical (QM) methods can be employed. These methods represent the most rigorous approach. They account for electronic transitions and coupling interactions within the peptide backbone. Unlike the previously described semiempirical methods, full QM approaches compute CD spectra directly from electronic wavefunctions or transition dipoles, thereby capturing complex phenomena such as exciton splitting. While computationally demanding, they provide the highest level of accuracy and are essential when precise spectral features are required to validate structural models [68, 69].

## 5 Additional Experimental Techniques that Can Support Molecular Dynamics Simulations: Small‐Angle Neutron Scattering/Small‐Angle X‐ray Scattering Spectroscopy

The difficulty of characterizing flexible multidomain proteins in solution frequently exceeds the capabilities of conventional high‐resolution techniques, such as X‐ray crystallography, which may exclude flexible regions or average out dynamic properties during structural refinement [70]. While MD simulations are crucial for generating the diverse conformational ensembles necessary to describe these flexible systems, the inherent limitations of FF and sampling methods mean that simulation results must be rigorously validated with experimental data. Methods previously used for comparison and validation of MD simulations in solution include nuclear magnetic resonance (NMR), small‐angle X‐ray scattering (SAXS), and small‐angle neutron scattering (SANS). NMR generally provides information regarding the relative orientation of atoms close in space, while SAXS is crucial because it carries information on the overall protein structure and global dimensions [71].

The comparison against SAXS data is particularly useful as a validation tool because it directly detects systematic errors in MD ensembles concerning global size and domain arrangement. For instance, studies on the protein TIA‐1 showed that MD simulations using the standard coarse‐grained Martini potential initially produced ensembles that were too compact, leading to pronounced discrepancies with SAXS profiles [70]. To achieve consistency after validation highlights an issue in the underlying FF, the ensemble is typically refined using approaches like the Bayesian maximum entropy method [70]. This refinement adjusts the conformational weights to achieve better consistency with the experimental constraints (such as SAXS data) while minimally perturbing the ensemble derived from the simulation. This framework, using MD to generate structural diversity and solution techniques like SAXS/SANS and NMR to validate and refine global and local parameters, constitutes a robust strategy for characterizing conformational heterogeneity [71].

## 6 Comparing Circular Dichroism Spectroscopy to Molecular Dynamics Simulation

### 6.1 Theory of Comparison

A rigorous comparison between CD measurements and MD simulations consists of a quantitative calculation of ellipticity from both experimental and theoretical approaches. On the experimental side, CD spectra are converted into mean residue ellipticity using standard equations that normalize for concentration, path length, and number of residues, ensuring that the measured signal reflects intrinsic secondary structure content rather than experimental conditions [18, 23].

On the computational side, the Hirst and Brooks model [56] provides a physically grounded route to compute ellipticity directly from MD structures by evaluating residue‐level electric dipole and magnetic dipole transition moments, their mutual orientations, and the excitonic couplings between peptide chromophores. Applied frame‐by‐frame across an equilibrated trajectory, this method generates an ensemble‐averaged ellipticity curve that is directly comparable to the experimental spectrum. We make a direction comparison of CD and MD below, such that the mean ellipticity at 222 nm can be quantified by either method and compared with Equation (4)

(4) $$\left[\theta \right]=\frac{100\cdot \left(\theta_{\mathrm{Obs}}\cdot 10^{-3}\right)\cdot M_{\mathrm{RW}}}{c\cdot l}≅\frac{V_{\infty }}{N_{r}}\sum_{i=1}^{N_{h}}w_{i}\left(r_{i}-k\right)$$

Within this integrated experimental–computational framework, a consistent definition of the quantities entering both approaches is essential. To this purpose, Table 1 summarizes the key parameters used in CD spectroscopy and MD‐based structural analysis, including experimental observables, normalization constants, and model‐dependent variables [19, 56, 72]. Particular emphasis is placed on the ellipticity at 222 nm, which serves as a widely adopted reporter of α‐helical content and thus provides a direct bridge between measured CD spectra and structure‐based MD predictions.

**TABLE 1:** Comparison of CD and MD. Parameters, units, and typical values used to calculate the mean residue ellipticity at 222 nm, ${\left[\theta \right]}_{222}$ from CD experiments and MD‐derived structural analysis. For the CD‐based calculation, ${\left[\theta \right]}_{222}$is obtained from the measured ellipticity at 222 nm using the mean residue molecular weight (*M*_RW_), protein concentration (*c*), and optical path length of the cuvette (*l*). For the MD‐based estimation, ${\left[\theta \right]}_{222}$ is computed from the α‐helical content, accounting for helix length, residue weighting, and an empirical correction parameter k that reflects reduced ellipticity contributions near helix endpoint. Typical values are provided for guidance [19, 56, 72]; protein‐specific parameters depend on sequence and structure of the molecule.

Symbol	Units	Typical values	Symbol	Units	Typical values
$\left[\theta_{222}\right]$	deg·cm^2^·dmol^−1^	−20,000 to −40,000 (α‐helix) ≈0 ± 2,000 (random coil) −5,000 to −10,000 (β‐sheet)	$\left[\theta_{222}\right]$	deg·cm^2^·dmol^−1^	−10,000 to −30,000 (α‐helix) ≈0 ± 2,000 (random coil)
$\theta_{222,\mathrm{CD}}$	millidegrees (mdeg)	−2,000 to −20,000 (α‐helix) ≈0 to −500 (random coil) −500 to −2,000 (β‐sheet)	$V_{\infty }$	deg·cm^2^·dmol^−1^	≈42.000
$M_{\mathrm{RW}}$	g·mol^−1^	Protein dependent	$N_{r}$	n° of residues	Protein dependent
c	g·mL^−1^	5.0 × 10^−5^ − 1.0 × 10^−3^	$w_{i}$	—	0−1
l	cm	0.01−1.0	$r_{i}$	—	Protein dependent
—	—	—	k	—	0 or 2.5 − 3

Integrating these two modes of ellipticity calculation explained by Equation (4), one from optical spectroscopy, the other from structural physics, enables a direct, wavelength‐resolved comparison between MD simulations and experiments. As shown in Table 1, simulations tend to underestimate absolute α‐helical ellipticity, likely due to incomplete sampling, force‐field bias, or simplifications in the Hirst and Brooks model such as neglecting solvent‐mediated effects.

Despite these differences, the overall trends are consistent: α‐helices are strongly negative, random coils near zero, and β‐sheets intermediate. Computing ${\left[\theta \right]}_{222}$ frame‐by‐frame along MD trajectories reproduces the dynamic ensemble properties measured by CD and provides basis for simulation validation.

We note that there are variants of CD spectroscopy that may be of interest to the reader. In particular, oriented circular dichroism is a technique that can measure the S and TM states of the helix in the oriented lipid bilayer [73, 74, 75].

### 6.2 Recommended Guidelines for Validation

In the previous section, we outlined the main equations to compare the α‐helical content estimated from CD and MD simulations. We now proceed to outline a set of community‐led best practices to ensure the optimal comparison of data from these techniques:

A.
*Optimize the sample quality and concentration* . High‐quality, pure samples are critical for CD measurements. Impurities or aggregation can distort spectra and lead to erroneous structural interpretations. Protein concentrations should be carefully selected to avoid excessive absorbance in the far‐UV region (typically 185–260 nm), which can reduce signal‐to‐noise ratio [19, 21].
B.
*Ensure instrument calibration and baseline correction* . Accurate CD data depend on a well‐calibrated instrument; baseline measurements of buffer alone must be performed and subtracted from sample spectra to correct for background signals, which prevents misinterpretation of secondary structure content [19, 21, 76].
C.
*Perform multiple measurements* . CD spectra are sensitive to experimental noise and small fluctuations. Collecting multiple scans of the same sample and averaging the results improves data reliability. In addition, measurements should be repeated under varying conditions (temperature, pH, etc.) when studying structural stability or conformational changes [19, 21, 76].
D.
*Use recent variants of CD spectroscopy where possible* . Oriented CD has proven very useful for the study of peptide partitioning through biological membranes [73, 74, 75].
E.
*Check your peptide* *FF* *carefully* . Whether you are employing CHARMM36, AMBER, or other choices, evaluate each on its own merit. The choice of FF can affect the folding properties of your peptide, as described in Section 3.
F.
*Ensure you sample the MD simulation process sufficiently long to guarantee convergence* . From a computational perspective, reliable application of Equation (3) requires a well‐equilibrated and sufficiently long MD trajectory, since *gmx helix* or *dssp* assignments are sensitive to hydrogen bond geometry and overall structural accuracy. Consequently, predicted [*θ*]_222_ values should be interpreted as ensemble‐averaged, force‐field‐dependent estimates that are most appropriate for qualitative or comparative assessment of peptide ellipticity rather than direct quantitative reproduction of experimental CD measurements.
G.
*The simulation setup choices matter* . The size of the simulation box and the ratio of water molecules and peptides/lipids will influence what is initially sampled in the simulation, and it is therefore recommended that the user carefully considers the relevant timescales of the simulation. Similarly, the lipid composition can impact the way the peptide interacts with the membrane, as has been discussed elsewhere [77]. We discussed a few examples of this in the main text.
H.
*When designing a novel peptide*, *use a neutral template to start with* . Chen and Ulmschneider [78] recommend using a polyleucine peptide as the initial design template to avoid introducing any bias with respect to the starting sequence. They argue that this is because leucine is the most abundant amino acid in MAPs and proteins [79, 80, 81], and they argue that polyleucine peptides form stable membrane‐spanning helices in lipid bilayers at lengths above 9 residues.
I.
*Follow FAIR principles for data sharing* [82]. Ensure that data are *findable*, *accessible*, *interoperable,* and *reproducible*. This applies both to the CD spectral data and MD data. According to best FAIR principles, use open‐source repositories to deposit your data such as Zenodo repository (http://zenodo.org/). This is increasingly a requirement by most journals and funders.

These guidelines highlight that a successful comparison between CD spectroscopy and MD simulations depends on rigorous and transparent practices across both experimental and computational workflows, including careful control of sample quality, measurement and simulation protocols, force‐field selection, and data management, to achieve explicable and reproducible findings.

## 7 Conclusions

In this work, we have outlined the primary applications of CD spectroscopy and MD simulations in the study of peptides interactions with biological membranes, highlighting their respective strengths and limitations. In particular, we discussed the key equations underlying each method, emphasizing how these formalisms can influence the interpretation of both experimental and computational data. Practical considerations were also addressed such as CD's sensitivity to peptide concentration and lipid composition, and, for MD, the importance of accurately choosing simulation timescales, FF parameters, size of simulation box, ratio of water molecules and lipids, and realistic membrane models.

We also provided a set of recommendations for comparing CD and MD datasets, emphasizing how an integrated approach can enhance both the reliability and depth of the insights obtained. Strategies for aligning experimental and computational results were discussed together with the inherent limitations of each method, thereby offering guidelines for a critical assessment of peptide–membrane interactions.

We envisage that this work can be used as a practical reference for researchers exploring the complementary strengths of CD and MD approaches. By integrating these methods, the community can gain more comprehensive insights into peptide mechanisms and guide the rational design of peptides with desired membrane‐targeting properties and membrane translocation properties.

## Author Contributions

**Cinzia Campus**: data curation (lead), formal analysis (lead), methodology (lead), writing – original draft (lead), writing – review & editing, (lead). **Marta Roldo**: conceptualization (equal), funding acquisition (equal), project administration (equal), writing – review & editing (equal). **Charles H. Chen**: investigation (equal), methodology (equal), writing – review & editing (equal). **Roja Hadianamrei**: conceptualization (equal), methodology (equal). **Elisabetta Gavini**: funding acquisition (equal), project administration (equal). **Christian Jorgensen**: conceptualization (lead), data curation supporting, formal analysis (equal), methodology (equal), project administration (equal), writing – original draft (equal), writing – review & editing (equal).

## Conflicts of Interest

The authors declare no conflicts of interest.

## Data Availability

Data sharing is not applicable to this article as no new data were created or analyzed in this study.
